# Characterisation of the pro-inflammatory cytokine signature in severe COVID-19

**DOI:** 10.3389/fimmu.2023.1170012

**Published:** 2023-03-30

**Authors:** Heike C. Hawerkamp, Adam H. Dyer, Neha D. Patil, Matt McElheron, Niamh O’Dowd, Laura O’Doherty, Aisling Ui Mhaonaigh, Angel M. George, Aisling M. O’Halloran, Conor Reddy, Rose Anne Kenny, Mark A. Little, Ignacio Martin-Loeches, Colm Bergin, Sean P. Kennelly, Seamas C. Donnelly, Nollaig M. Bourke, Aideen Long, Jacklyn Sui, Derek G. Doherty, Niall Conlon, Cliona Ni Cheallaigh, Padraic G. Fallon

**Affiliations:** ^1^ School of Medicine, Trinity Biomedical Sciences Institute, Trinity College Dublin, Dublin, Ireland; ^2^ Department of Medical Gerontology, School of Medicine, Trinity College Dublin, Dublin, Ireland; ^3^ Department of Age-Related Healthcare, Tallaght University Hospital, Dublin, Ireland; ^4^ Department of Infectious Diseases, St James’s Hospital, Dublin, Ireland; ^5^ Trinity Kidney Centre, Trinity Translational Medicine Institute, Trinity College, Dublin, Ireland; ^6^ Department of Intensive Care Medicine, St James’s Hospital, Dublin, Ireland; ^7^ Department of Clinical Medicine, Trinity Translational Medicine Institute, Trinity College Dublin, Dublin, Ireland; ^8^ Department of Clinical Medicine, Tallaght University Hospital, Dublin, Ireland; ^9^ Trinity Translational Medicine Institute, Trinity College Dublin, Dublin, Ireland; ^10^ Department of Immunology, St James’s Hospital, Dublin, Ireland; ^11^ Department of Immunology, Trinity Translational Medicine Institute, Trinity College Dublin, Dublin, Ireland; ^12^ St James’s Hospital, Tallaght Hospital, Wellcome Trust Health Research Board Clinical Research Facility, Dublin, Ireland

**Keywords:** COVID-19, SARS-CoV-2, inflammation, cytokine, plasma

## Abstract

Clinical outcomes from infection with SARS-CoV-2, the cause of the COVID-19 pandemic, are remarkably variable ranging from asymptomatic infection to severe pneumonia and death. One of the key drivers of this variability is differing trajectories in the immune response to SARS-CoV-2 infection. Many studies have noted markedly elevated cytokine levels in severe COVID-19, although results vary by cohort, cytokine studied and sensitivity of assay used. We assessed the immune response in acute COVID-19 by measuring 20 inflammatory markers in 118 unvaccinated patients with acute COVID-19 (median age: 70, IQR: 58-79 years; 48.3% female) recruited during the first year of the pandemic and 44 SARS-CoV-2 naïve healthy controls. Acute COVID-19 was associated with marked elevations in nearly all pro-inflammatory markers, whilst eleven markers (namely IL-1β, IL-2, IL-6, IL-10, IL-18, IL-23, IL-33, TNF-α, IP-10, G-CSF and YKL-40) were associated with disease severity. We observed significant correlations between nearly all markers elevated in those infected with SARS-CoV-2 consistent with widespread immune dysregulation. Principal component analysis highlighted a pro-inflammatory cytokine signature (with strongest contributions from IL-1β, IL-2, IL-6, IL-10, IL-33, G-CSF, TNF-α and IP-10) which was independently associated with severe COVID-19 (aOR: 1.40, 1.11-1.76, p=0.005), invasive mechanical ventilation (aOR: 1.61, 1.19-2.20, p=0.001) and mortality (aOR 1.57, 1.06-2.32, p = 0.02). Our findings demonstrate elevated cytokines and widespread immune dysregulation in severe COVID-19, adding further evidence for the role of a pro-inflammatory cytokine signature in severe and critical COVID-19.

## 1 Introduction

Infection with SARS-CoV-2, the cause of the COVID-19 pandemic, has resulted in over 6 million deaths since December 2019 (1). COVID-19 outcomes remain remarkably variable, ranging from asymptomatic infection to severe pneumonia and death. Clinical risk factors for critical COVID-19 include male gender, obesity, age, frailty, chronic medical comorbidities and immunodeficiency states (2, 3). Whilst an unprecedented effort has resulted in crucial insights into the immunopathogenesis of severe COVID-19, individual trajectories in immune response remain to be further elucidated which holds the promise of personalised treatment for the dysregulated immune response in COVID-19, as well as more generally broadening our understanding of the immune response to other severe infections and critical illness states.

In those infected SARS-CoV-2, COVID-19 disease typically follows a biphasic pattern (4, 5). The first phase involves viral replication and direct virus-mediated tissue damage, with recruitment of effector cells resulting in both local and systemic inflammatory responses in the second phase. Severe COVID-19 is associated with a profound lymphopenia, defective Th1 and excess Th2 immune responses, impairing effective clearance of virus (6). Autopsy studies demonstrate that hyper-inflammation and the inability to control SARS-CoV-2-driven inflammatory responses are associated with organ failure and critical COVID-19. An excess in circulating monocytes, neutrophils and myeloid progenitors (termed “emergency myelopoesis”) has also been well documented as a critical feature of severe COVID-19 (7, 8).

As SARS-CoV-2 undergoes replication within infected epithelial cells of the upper respiratory tract and lungs, it is detected by innate immune cells such as plasmacytoid Dendritic Cells (pDCs) (4). Detection of SARS-CoV-2 by Pathogen Recognition Receptors (PRR) such as endosomal Toll-Like Receptors (TLRs) and RIG-I-like receptors results in activation of both the type I interferon (IFN) and nuclear factor kB (NF-kB) responses resulting in the production of pro-inflammatory cytokines and chemokines (4). The important role of appropriate activation and tight regulation of this early innate type I IFN response is supported by associations between genetic variants in the type I IFN response/autoantibodies inhibiting the type I IFN response and severe/critical COVID-19 (9–11). Additionally, the importance of NF-kB-dependent cytokine and chemokine production in severe/critical COVID-19 has been consistently reported – with many studies examining Interleukin (IL)-6 in plasma as a critical predictor of COVID-19 severity and mortality (12–15). Further, the use of the IL-6 inhibitor Tocilizumab is now known to improve survival in hospitalised severe COVID-19 patients with systemic inflammation (16–19).

A number of studies have demonstrated the association between a hyper-inflammatory state, severe disease and mortality from COVID-19. Apart from the most commonly studied inflammatory markers – namely IL-6/CRP, other immune markers which have demonstrated associations with severe COVID-19 include IL-1 family (IL-1β, IL-1RA, IL-18, IL-33) (20), Th1-related (IL-2, IL-2R, IL-12, IL15, IL-27, TNFα, sTNFR1) (21), Th2-related (IL-4, IL-5, IL-10, IL-13) (22) and Th17-related (IL-17A, IL-22) (23) cytokines, chemokines (IL-8, MCP-1, MCP-3, MIP-1α, MIP-1β, MIP-3β, CXCL9) (24) interferon-related (IFN-y, IP-10) proteins and growth factors (IL-7, TGF-β, GM-CSF, G-CSF) (25) - although results are heterogenous and in many cases limited by small sample sizes of clinical cohorts (26–29). Studies have additionally demonstrated the utility of cytokine ratios (namely IL-6:IL-10) in predicting severe/critical COVID-19 (30). Combinations of cytokines have also been explored, most commonly the quartet of IL-6, IL-8, IL-1β and TNFα due to widespread availability on several multiplexing platforms. One of the most comprehensive studies to date assessed plasma levels of these four cytokines in 1,484 hospitalised patients with COVID-19 and found robust associations between IL-6, IL-8 and TNFα levels and mortality – a finding widely replicated (16, 25, 31, 32).

In the current study, we used a comprehensive series of multiplexing assays, including an ultra-sensitive assay for analytes typically present at low levels in plasma, to assess 20 circulating inflammatory plasma biomarkers in 118 vaccine-naïve individuals with COVID-19 recruited during the first year of the pandemic and 44 SARS-CoV-2 uninfected healthy controls to characterise the acute inflammatory response in individuals with COVID-19 and examine whether this response was associated with important clinical outcomes in acute COVID-19.

## 2 Methods

### 2.1 Study participants and recruitment

This study was carried out within the St James’s Hospital, Tallaght University Hospital, Trinity College Dublin Allied Researchers’ (STTAR) Bioresource based in Dublin, Ireland which has been previously described (33). The STTAR Bioresource is a dual-site cohort study across two university teaching hospitals which has recruited patients with Polymerase Chain Reaction (PCR) confirmed acute SARS-CoV-2 infection (Cobas ^©^ SARS-CoV-2 test, Roche Diagnostics, Rotkreuz, Switzerland) in addition to healthy control groups. Written informed consent was obtained by the study team and study participation occurred in line with the Declaration of Helsinki. Full ethical approval for the STTAR Bioresource was granted by the St James’s Hospital/Tallaght University Hospital Joint Research and Ethics Committee (JREC 2020-05 List 19).

For the current study a representative sample of participants were taken from the wider Bioresource cohort representing those with mild, moderate and severe COVID-19 in addition to SARS-CoV-2 naïve healthy controls (whom were either unvaccinated or >4 weeks post primary vaccine course) free from significant medical comorbidity. All COVID-19 participants were diagnosed with PCR-confirmed COVID-19 between October 2020-January 2021 and were unvaccinated. Blood samples were taken in the morning, generally following diagnosis or admission alongside routine phlebotomy. Fresh blood samples were processed on the same morning, with samples centrifuged at 1,400 *x g* at room temperature for 10 minutes and plasma aliquoted and stored at -80°C for future analysis. Routine haematological and biochemical laboratory results were obtained at the earliest timepoint from the first positive PCR test.

### 2.2 Clinical data and assessment of COVID-19 severity

Clinical data collected by the study team includes demographic information (age, sex, level of education, smoking status) and background medical history. Premorbid frailty status was classified by the study team based on the Clinical Frailty Score (CFS) (34). With regards to acute COVID-19, initial presenting symptoms are recorded from each participant in addition to date of symptom onset, date of positive PCR test, oxygen requirement at presentation and throughout admission, receipt of intravenous/oral steroids, medications for COVID-19 and length of stay in hospital.

To assess COVID-19 severity, the World Health Organisation (WHO) Clinical Progression Scale was used, in line with other clinical cohort studies on acute COVID-19 (35). This scale reflects clinical trajectory and resource use over the course of acute COVID-19. This is a ten-point scale and was used to classified individuals into disease severity categories as follows: (i) Mild Disease (Score 1-3): ambulatory individuals who were asymptomatic or symptomatic, independent or with assistance needed, (ii) Moderate Disease (Score 4-5): those hospitalised for COVID-19 with no oxygen therapy needed or oxygen therapy required *via* mask or nasal prongs (iii) Severe Disease (Score 6-9): patients hospitalised with a need for oxygen by non-invasive ventilation/high flow, those needing intubation and mechanical ventilation, with or without vasopressors/dialysis/extra-corporeal membrane oxygenation, (iv) Dead (Score 10). The severity score for each participant represents the maximum severity score for each participant through the course of acute COVID-19.

### 2.3 Analysis of immune markers using high-sensitivity multiplex assays

To characterise the immune response in acute COVID-19, a panel of 20 immune-related biomarkers were designed based on a comprehensive literature review. The final panel included the NF-kB-dependent IL-6, Th1-related (IL-2, IL-12p70, TNFα), Th2-related (IL-4, IL-5, IL-10, IL-13), Th17-related (IL-17A, IL-23) and IL-1 family (IL-1β, IL-18, IL-33), cytokines, chemokines (MCP-1, MIP-1β), interferon-related (IFN-y, IP-10) proteins, growth factors (TGF-β, G-CSF) and YKL-40. A custom U-Plex Enzyme-Linked Immunosorbent Assay (ELISA) kit was designed (U-PLEX Human Biomarker Kit, Meso Scale Diagnostics, MSD, MD, USA), spread across four discrete assays. Assays were conducted as per the manufacturers’ instructions with plates read using a MESO QuickPlex SQ 120MM instrument. Protein concentrations (in pg/mL) were calculated using Meso Scale Diagnostics Discovery Workbench Software (v4.0). Several analytes had a high frequency of undetectable concentrations in plasma of both COVID-19 patients and controls and these 8 analytes were re-analysed (namely IL-1β, IL-2, IL-4, IL-6, IL-10, IL-12p70, IL-17A, TNF-α and IFN-y) using an ultra-sensitive assay (S-PLEX, Meso Scale Diagnostics, MSD, MD, USA) and read on the same platform enabling quantification of immune marker concentration in femtograms. For participants with undetectable levels of analytes in serum, these were replaced by the Lower Limit of Detection (LLOD) for subsequent analysis.

### 2.4 Statistical analysis

Between-group differences between COVID-19 patients and healthy controls in baseline/disease characteristics and immune marker levels were analysed in the first instance using t-tests, wilcoxon ranks-sum and chi-square tests as appropriate. For comparison of baseline/disease characteristics and immune marker levels across disease severity groupings (mild/moderate/severe), ANOVA and Kurskal-Wallis tests were used with a *post-hoc* Dun’s test. For correlational analysis, Spearman rank tests/Pearson’s test were used as appropriate and a Bonferrroni correction applied for multiple testing. For analysis of correlations and principal components, cytokine data was natural log-transformed and expressed as z-scores of the overall cohort due to the strong right skew within nearly all analytes.

Principal Component Analysis (PCA) was subsequently performed on all analytes which significantly differed across severity groups for individuals with COVID-19. Data was first examined using the Kaiser-Meyer-Olkin measure and the Bartlett test of sphericity to evaluate the suitability of the dataset for PCA. PCA was performed using only the immune markers which were associated on univariate analysis with COVID-19 severity and was performed on COVID-19 patients only in order to explain the variability within those with acute COVID-19. Principal Component scores for components with an Eigenvalue > 1.0, consistent with the Kaiser-Guttman criteria, were retained and individual scores for these components assigned to those with acute COVID-19.

Logistic regression was used to assess the relationship between components and the following outcomes: (i) severe disease, (ii) need for invasive mechanical ventilation and (iii) mortality, with models adjusted for age, sex, dexamethasone use and days from first symptom to blood sampling as covariates. Results of logistic regression are presented as adjusted Odds Ratios (aOR) with 95% Confidence Intervals (CI) as appropriate. Across all analysis, a p-value of <0.05 was considered statistically significant. Data analysis was performed on RStudio and STATAv17.0 whilst data visualisation was performed using GraphPadv9.0 and “ggplot2”/”FactoMineR” packages on RStudio.

## 3 Results

### 3.1 Participant cohort

In the study 118 individuals (median age: 70, IQR: 58-79 years; 48.3% female) with acute SRAS-CoV-2 infection were included. As per the above WHO criteria, just over one-fifth (N = 26, 22%) had mild COVID-19, whilst half (N = 58, 49.2%) had moderate and the remainder (N = 34, 28.8%) had severe COVID-19 (**Table 1**). There were no significant differences in age, sex, medical comorbidity (prevalence of chronic obstructive pulmonary disease/asthma/type 2 diabetes/cardiac disease or malignancy) or frailty status across disease severity groups (all p>0.05). Just over two-thirds (23/34; 67.7%) of individuals with severe COVID-19 as classified by the WHO severity score were admitted to the Intensive Care Unit. Overall 30-day mortality due to severe COVID-19 in the cohort was 10.2% (12/118) (**Table 1**). Consistent with previous reports, participants with severe disease had a greater oxygen requirement, treatment with dexamethasone and elevated White Cell Count, Neutrophil Count, Neutrophil : Lymphocyte Ratio and C-Reactive Protein (CRP) on routine blood tests. Apart from dexamethasone, no participants in the current study were treated with Remdesivir or Tocilizumab and all with acute COVID-19 were unvaccinated. **Table 1** provides a detailed breakdown of demographic, medical history and acute illness characteristics.

**Table 1:** Characteristics of participants (N = 118) by COVID-19 acute illness severity.

Characteristic	Mild (*N = 26; 22.0%)* *WHO Score 1-3*	Moderate (*N = 58; 49.2%)* *WHO Score 4-5*	Severe (*N = 34; 28.8%)* *WHO Score 6-10*	Statistic
Age in Years	67.2 (18.6)	65.5 (17.4)	72.5 (12.7)	F = 2.66, p=0.08
Sex, Female	17 (65.4%)	28 (48.3%)	12 (35.3%)	χ^2^ = 5.3, p=0.07
Smoking Status *Current* *Former* *Never*	2 (8.3%) 6 (25%) 16 (66.7%)	2 (3.5%) 23 (49.7%) 33 (56.9%)	3 (8.8%) 9 (26.5%) 22 (64.7%)	χ^2^ = 3.4, p=0.50
Medical Comorbidity *COPD* *Asthma* *Type 2 Diabetes* *Cardiac Disease* *Malignancy (Past/Current)*	4 (15.4%) 1 (3.5%) 6 (23.1%) 7 (26.9%) 0 (0%)	12 (20.7%) 9 (15.5%) 10 (17.2%) 14 (24.1%) 5 (8.6%)	3 (8.8%) 4 (11.9%) 7 (20.6%) 10 (29.4%) 4 (11.8%)	χ^2^ = 2.3, p=0.33 χ^2^ = 2.3, p=0.31 χ^2^ = 0.4, p=0.81 χ^2 =^ 0.3, p=0.85 χ^2 =^ 3.1, p=0.22
Clinical Frailty Score	3 (1-6)	2 (1-4.5)	3 (2-5)	χ^2 =^ 2.2, p=0.32
COVID-19 Presenting Symptoms *Fever* *Cough* *Dyspnoea* *Fatigue/Malaise* *Anorexia* *Diarrhoea* *Nausea/Vomiting* *Myalgia/Joint Pain*	6 (23.1%) 13 (50%) 11 (42.3%) 4 (15.4%) 2 (7.7%) 2 (7.7%) 3 (11.5%) 3 (11.5%)	25 (43.1%) 39 (67%) 38 (65.5%) 15 (25.9%) 8 (13.8%) 6 (10.3%) 12 (20.7%) 9 (15.5%)	11 (32.4%) 20 (58.8%) 24 (70.6%) 14 (41.2%) 9 (26.5%) 7 (20.6%) 6 (17.7%) 7 (20.6%)	χ^2 =^ 3.4, p=0.19 χ^2 =^ 2.3, p=0.31 χ^2 =^ 5.6, p=0.06 χ^2 =^ 5.1, p=0.08 χ^2 =^ 4.3, p=0.12 χ^2 =^ 2.8, p=0.25 χ^2 =^ 1.0, p=0.60 χ^2 =^ 0.9, p=0.63
COVID-19 Illness *Admitted for COVID-19 Care* *Intensive Care Admission Required* *Required Supplemental Oxygen* *Peak Oxygen Requirement (Fi02)* *Treated with Dexamethasone*	4 (15.4%) 0 (0%) 0 (0%) 0.21 (0.21-0.21) 3 (11.5%)	51 (87.9%) 0 (0%) 45 (77.6%) 0.28 (0.24-0.36) 46 (79.3%)	34 (100%) 23 (67.7%) 34 (100%) 0.78 (0.4-1) 30 (88.2%)	χ^2 =^ 66.5, p<0.001 χ^2 =^ 70.6, p<0.001 χ^2 =^ 72.4, p<0.001 χ^2 =^ 74.5, p<0.001 χ^2 =^ 47.1, p<0.001
Laboratory Values At Assessment *Days From Symptom Onset to Blood Draw* *White Cell Count* *Neutrophils* *Lymphocytes* *Neutrophil : Lymphocyte Ratio* *Monocytes* *C Reactive Protein*	6 (3-8) 5.2 (4-6.3) 3.3 (2.1-4.2) 1.2 (0.9-1.6) 2.7 (2-4.1) 0.5 (0.4-0.6) 10.6 (6.8-44.8)	8 (5-12) 6.4 (4.7-9.2) 4.4 (3.1-6.9) 1.1 (0.8-1.7) 4 (2.8-6.9) 0.5 (0.3-0.8) 41.6 (9.0-71.8)	7 (4-11) 9.4 (5.6-14.7) 8.7 (4.6-12.3) 0.8 (0.5-1.4) 9.9 (5-13.5) 0.5 (0.3-0.9) 72.6 (44.4-128.1)	χ^2 =^ 3.5, p=0.18 χ^2 =^ 13.8, p<0.001 χ^2 =^ 21.6, p<0.001 χ^2 =^ 5.7, p=0.06 χ^2 =^ 30.2, p<0.001 χ^2 =^ 0.3, p=0.88 χ^2 =^ 16.1, p<0.001
Inpatient Admission *Length of Stay* *30-Day Inpatient Mortality*	- 0 (0%)	12.5 (6.5-31.5) 0 (0%)	32 (21-40) 12 (35.3%)	χ^2 =^ 8.4, p=0.02 χ^2 =^ 33.0, p<0.001

Data are presented as mean and standard deviation or median with interquartile range as appropriate. Count data are presented as numbers and percentages. ANOVA and Kruskal-Wallis tests were used to analyse differences in continuous data between severity groups for parametric and non-parametric data respectively, whilst chi-square tests were used for categorical data. -, data not applicable.

All 118 participants in the current cohort donated a plasma sample during their acute illness. The median time from first COVID-19 symptom (or date of positive PCR in asymptomatic individuals) to sample donation was 7 days (IQR: 4-11) which did not significantly differ across the three disease severity categories (χ^2 =^ 3.5, p=0.18, Kurskal-Wallis test, **Table 1**). Samples included were obtained at time of maximal disease severity for mild/moderate/severe disease. Of 79 individuals treated with dexamethasone, the vast majority (93.7%; 74/79) were on treatment for at least 24 hours at time of blood draw. Cytokine levels were compared to a convenience cohort of SARS-CoV-2 naïve healthy controls whom were significantly younger (48.0 +/- 15.5 for controls vs 66.5 +/-16.7 for COVID-19 participants, t = -6.3, p<0.001) with a greater proportion of females (65% female for controls vs 48% for COVID-19 participants, χ^2 =^ 4, p<0.05). Two-fifths of the healthy control group (18/44, 40.9%) were at least 4-weeks post primary SARS-CoV-2 vaccination course (15/18, 83.3% received the Pfizer BioNTech mRNA vaccine whilst 3/18, 16.7% received a primary vaccine course with the AstraZenica vaccine). The remainder were unvaccinated at time of recruitment.

### 3.2 Cytokine response in acute COVID-19

**Figure 1:**
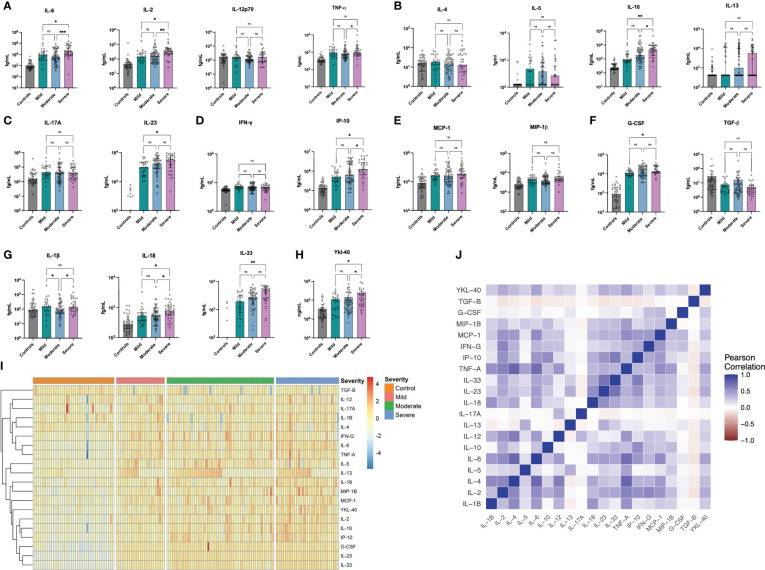
Cytokine Concentrations in Acute COVID-19. IL-1β, IL-4, IL-5 and IL12p70 were not significantly elevated in acute COVID-19 patients whilst all other cytokines were significantly elevated (TGF-B was significantly lower in acute COVID-19). Eleven Markers, namely IL-1β, IL-2, IL-6, IL-10, IL-18, IL-23, IL-33, TNF-α, IP-10, G-CSF and YKL-40 all differed significantly by COVID-19 Severity. Data are presented for controls (n = 44) and those with mild (n = 26), moderate (n = 58) and severe (n = 44) COVID-19 for NF-kB-dependent IL-6 & Th1 cytokines **(A)**, Th2 cytokines **(B)**, Th17 cytokines **(C)**, interferon-related proteins **(D)**, chemokines **(E)**, growth factors **(F)**, IL-1 family cytokines **(G)** and Ykl-40 **(H)**. Results are presented for A-H in femtograms/mL (fg/mL) on a logarithmic scale. Results are also presented as a heatmap of log-transformed, z-scored concentrations based on disease severity for the entire cohort **(I)** and as a correlation matrix **(J)** demonstrating the relationships between differing markers. *p<0.05, **p<0.01, ***p<0.001, ns, non-significant. Strongest correlations were seen (R>0.7) between 6 pairs: IL-1β & IL-4, IL-1β & TNF-α, IL-1β & IL-6, between IL-6 & TNF-α; between TNF-α and IL-10 and between IP-10 & IL-33 which all persisted following Bonferonni correction.

Individuals with acute COVID-19 had significantly elevated levels of 16 of the 20 markers studied - namely IL-2, IL-5, IL-6, IL-10, IL-13, IL-17A, IL-18, IL-23, IL-33, TNF-α, IP-10, IFN-y, MCP-1, MIP-1β, G-CSF and YKL-40 - with significantly lower levels of TGF-β in comparison to healthy controls. The greatest differences were seen for IL-2, IL-6, IL-10, TNF-α, IP-10 and G-CSF (all p<0.001, **Table S1**). Amongst those with acute COVID-19, eleven markers significantly differed across disease severity categories: IL-1β, IL-2, IL-6, IL-10, IL-18, IL-23, IL-33, TNF-α, IP-10, G-CSF and YKL-40 (**Table S2**, **Figure 1**). Significant differences were seen for severe vs moderate disease in levels of eight markers (IL-1β, IL-2, IL-6, IL-10, IL-18, IP-10, TNF-α and YKL-40) whilst for severe vs mild, elevations were seen for nine markers (IL-2, IL-6, IL-10, IL-18, IL-23, IL-33, IP-10, YKL-40 and G-CSF) (**Figures 1A–H**). Within the severe disease category, there were no significant differences between in inflammatory markers between those on high flow oxygen (WHO Severity Score: 6), those who had Invasive Mechanical Ventilation (WHO Severity Score: 7-9) and those who died (WHO Severity Score: 10) for any of the above analytes (**Figure S1**).

**Figure 2:**
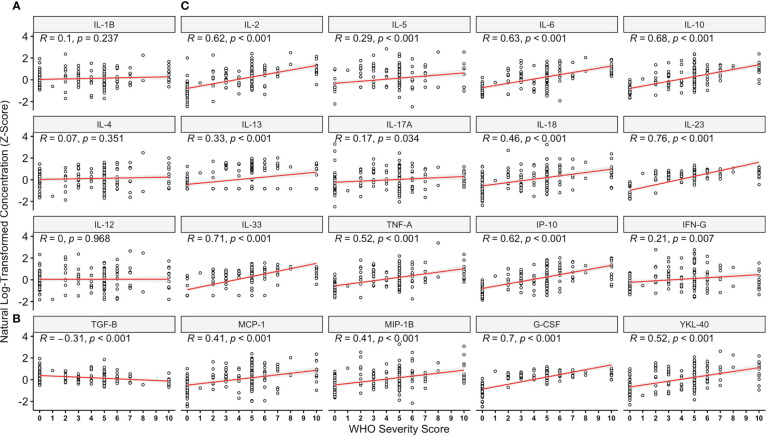
Relationships Between Analytes Studied and World Health Organisation Disease Severity Score. On analysing immune marker concentration (z-score), and maximal disease severity rated using the World Health Organisation (WHO) COVID-19 severity score, there were no associations observed for IL-1β, IL-4 or IL-12p70 **(A)** whilst a negative association was seen for TGF-β **(B)**. All remaining 16 markers demonstrated significant correlations with WHO severity score **(C)**, with the strongest associations observed for IL-23, IL-33, G-CSF, IL-6, IL-2 and IL-10. Data are presented for the entire cohort including controls (n = 44, WHO score = 0) and those with acute COVID-19 illness (n = 118, WHO score = 1-10).

On analysing all pairwise comparisons amongst individuals with acute COVID-19, most cytokines exhibited low-moderate correlation (see **Figure 1**). Strong correlations (R > 0.7) were observed for six pairs, namely (i) IL-1β & IL-4, (ii) IL-1β & TNFα, (iii) IL-1β & IL-6, (iv) IL-6 & TNF-α, (v) TNF-α & IP-10 and (vi) IP-10 & IL-33 (all p<0.001) which persisted following Bonferonni correction for multiple testing. A full correlation matrix is provided in the Supplementary Materials (See **Table S3**) and presented graphically in **Figure 1**. On analysing the correlation between overall marker concentration and COVID-19 WHO severity score (ranging from 1-10), there were no associations observed for IL-1β, IL-4 or IL-12p70 (all p>0.05, **Figure 2A**) and a negative association was seen for TGF-β (p<0.001, **Figure 2B**). All of the remaining 16 markers demonstrated significant correlations with WHO severity score, with the strongest associations observed for IL-23, IL-33, G-CSF, IL-6, IL-2 and IL-10 (all p<0.001, **Figure 2**).

### 3.3 Principal component analysis (PCA) of cytokine response

**Figure 3:**
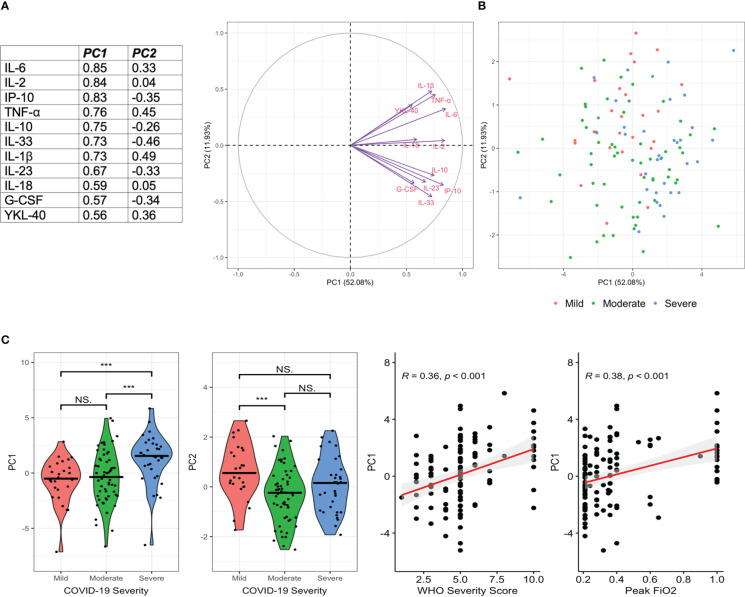
Principal Components Analysis Demonstrates a Pro-Inflammatory Cytokine Signature in Severe COVID-19. Principal Components Analysis (PCA) was performed in those with acute COVID-19 to characterise the variability in inflammatory markers within this cohort. 11 markers which differed by disease severity category were included. PC1 and PC2 explained 52.08% and 11.93% of the variance respectively. Markers contributing most to PC1 included IL-1β, IL-2, IL-6, IL-10, IL-33, G-CSF, TNF-α and IP-10 **(A)**. Individual PC1 and PC2 values are given by disease severity **(B)**. PC1 was significantly higher in severe disease, whilst PC2 was significantly lower in moderate than mild disease **(C)**. PC1 was significantly correlated with WHO Severity Score and increasing oxygen requirements **(C)**. ***p<0.001, ns, non-significant.

Given the association of multiple inflammatory markers with COVID-19 severity, we employed PCA to further evaluate variability within the 11 markers which significantly differed across COVID-19 severity categories. PCA resulted in two components with an Eigenvalue >1.0 which explained 64% of the variance in these 11 markers in participants with COVID-19 (PC1 accounted for 52.08% of the variance whilst PC2 accounted for 11.93%) (**Figure 3A**). The markers with the greatest contribution to PC1 included IL-2, IL-6, IP-10, TNF-α, IL-10, IL-33 and IL-1β. When assessed across disease severity groupings, PC1 was significantly higher in those with severe disease vs mild and severe vs moderate COVID-19 (both p<0.001, **Figure 3B**), but did not significantly differ in those with mild vs moderate COVID-19. PC2 was significantly lower in moderate vs mild COVID-19 (**Figure 2**).

Using logistic regression models with adjustment for age, sex, dexamethasone use and days since symptom onset, greater scores on PC1 were independently associated with severe disease (aOR: 1.40, 1.11-1.76, p=0.005), need for invasive mechanical ventilation (aOR: 1.61, 1.19-2.20, p = 0.001) and mortality (aOR 1.57, 1.06-2.32, p = 0.02). PC2 was not associated with any of these outcomes.

## 4 Discussion

In the current study, we analysed 20 immune markers in plasma from 118 unvaccinated individuals with acute COVID-19 during the first year of the pandemic. Acute COVID-19 was associated with a significant elevation in pro-inflammatory cytokines with eleven markers being associated with disease severity categories (namely IL-1β, IL-2, IL-6, IL-10, IL-18, IL-23, IL-33, TNF-α, IP-10, G-CSF and YKL-40).Many of the markers were highly correlated and reflect the known hyper-inflammatory phenotype of severe COVID-19 (36). Whilst two components derived from PCA on the COVID-19 patient cohort (**Figure 3**) explained 52.1% and 11.9% of the variance respectively, contributions to both components reflected widespread immune dysregulation and a severe pro-inflammatory phenotype. In particular, PC1 reflected the known hyper-inflammatory phenotype of COVID-19 (with greatest contributions from IL-2, IL-6, IP-10, TNF-α, IL-10, IL-33 and IL-1β) which was independently associated with severe disease, requirement for invasive mechanical ventilation and mortality.

The largest cluster identified by PCA reflects the widespread immune dysfunction present in severe COVID-19 and confirms previous reports supporting the role of many of these markers individually. In particular, many reports have focused on IL-6, TNF-α, IL-1β and IL-10 whilst comparatively fewer have evaluated IP-10, G-CSF and IL-33. Interestingly, some early studies reported associations between IP-10 levels and both disease progression and mortality in COVID-19 (37, 38). Importantly IP-10 may be an important mediator of lung injury in acute COVID-19 (39), and a single observational study which performed hierarchical assessment of 53 markers found IP-10 to be the marker most significantly associated with COVID-19 outcome (40). Meanwhile for G-CSF, elevated levels have been associated with greater oxygen requirement, disease severity and mortality in COVID-19 (13, 28, 41). Several studies have reported elevated IL-33 in individuals with COVID-19, with levels increasing with greater disease severity (20, 22, 42–44). IL-33 producing cells have even been isolated in bronchoalveolar lavage fluid in individuals with severe COVID-19 pneumonia (45). Our findings confirm a defined cytokine signature in severe COVID-19 (with elevated IL-2, IL-6, TNFα, IL-1β and IL-10), and add further evidence to the role of IP-10, G-CSF and IL-33 in the cytokine milieu associated with severe COVID-19.

One of the earliest and most robust findings in severe/critical COVID-19 patients has been the development of elevated circulating pro-inflammatory cytokines indicating immune hyper-responsiveness. “Cytokine Storm Syndrome” (CSS) is an umbrella term encompassing several disorders of immune dysregulation characterised by systemic inflammation and multi-organ dysfunction (46). Whilst early reports labelled the elevated cytokine levels in COVID-19 as CSS, and further asserted CSS as a key mechanistic driver of critical COVID-19, other studies noted lower levels of circulating cytokines in COVID-19 than other pathologies such as Cytokine Release Syndrome (CRS) and severe sepsis (47–50). This means that the inflammatory cytokine milieu in COVID-19 may be more distinctive than classical CSS and also be unique in comparison to other respiratory viruses (36, 51). Understanding hyper-inflammatory phenotypes and trajectories in severe/critical COVID-19, as described herein, may enable personalised approaches to immunosuppression targeted at the hyper-inflammatory immune response seen in some - but not all - individuals with severe COVID-19 (52). Previous reports have noted the differing levels of cytokine elevation in COVID-19. Whilst initial reports of CSS in severe/critical COVID-19 were debated, many subsequent studies reframed the levels of circulating pro-inflammatory cytokines as a moderate cytokine elevation (53) in comparison to other conditions such as CRS. Our findings in the current study further support the evidence for moderate cytokine inflammation in COVID-19 at consistent levels with other international cohorts, much lower than seen in CRS (47). This is most seen for IL-6 – the most widely studied cytokine in acute COVID-19 - where using a highly sensitive assay reporting results to the femtogram, we see similar results across disease severity categories – with levels much lower than that typically seen in CRS.

Our study has several notable strengths. We analysed plasma samples of individuals hospitalised during the first year of the pandemic and thus no participants in the current study had received vaccination. This provides a unique insight into the immune response to SARS-CoV-2 and in COVID-19 in an antigen-naïve cohort. Thus, the cohort under study represent a unique opportunity to profile the dysregulated cytokine response in severe COVID-19 in those without prior exposure to the virus. Whilst two-fifths of the control group had been vaccinated, none of those in the COVID-19 cohort had been, again providing a unique opportunity to profile the unique cytokinemia associated with severe and critical COVID-19. These findings confirm previous reports and they add to the significant evidence base supporting widespread immune dysregulation in severe and critical COVID-19 in an SARS-CoV-2 naïve cohort. Our cohort was largely representative of other studies within hospital populations, with a comparable number experiencing severe COVID-19 and a similar mortality rate. Thus, our findings have wider applicability in the association between a distinct cytokine profile and severe/critical COVID-19.

One of the significant challenges in interpreting cytokine data in those with COVID-19 centres around laboratory and assay variability (54). Further, many cytokines which may be important in the pathogenesis may be undetectable in the blood of COVID-19 patients, necessitating the use of highly sensitive assays for their quantification. The association between elevations in a cytokine in one laboratory but not in another has important implications for our understanding not only of COVID-19 pathogenesis but also therapeutic choices (54, 55). In the current study, we used a highly sensitive multiplex platform to quantify circulating cytokines in plasma. For those cytokines with low expression levels, typically not detected in plasma, we used a more sensitive assay, reporting results in femtograms, enabling further characterisation of the cytokine signature in severe COVID-19.

There are some limitations in the current study worthy of note. Nearly all patients were treated with dexamethasone, which has known potent effects on the immune response to acute COVID-19 with potent effects on neutrophil differentiation and function (56), although effects on serum variation of biomarkers has been limited in some studies (43). In the current study, we were unable to determine the impact of dexamethasone on immune response trajectories, although this has been widely studied. Importantly, none of the individuals in the current study were treated with other immunosuppressive agents such as Tocilizumab with known effects on circulating cytokines as a direct result of their mechanism of action (57). Further, our study is a single site study and so findings may be limited beyond the current cohort. Nevertheless, these results are consistent with previous studies characterising the distinct cytokine signature in COVID-19 and adds further evidence to a defined pro-inflammatory cytokine signature in COVID-19 which is associated with severe disease, need for invasive mechanical ventilation and mortality.

There are still several unanswered questions surrounding the cytokine signature in acute COVID-19. One of the most important centres around the temporal association between immune markers and how these relate to disease trajectories and outcomes. For instance, recent studies have demonstrating that the timing when cytokines are measured may be critical in understanding the immune response in acute COVID-19 (58). Further understanding which markers are elevated at each stage of the disease and how this relates to COVID-19 outcomes is an important area for future research in identifying different inflammatory phenotypes for targeted therapy in acute COVID-19. Approaches such as point-of-care testing for inflammatory markers and detailed characterisation of the immune response in COVID-19 may allow targeted treatment for different immune sub-phenotypes in both severe COVID-19 other critical illness states (59).

## 5 Conclusion

We studied the dysregulated cytokine signature in 118 individuals with acute COVID-19. Severe COVID-19 was associated with significantly elevated levels in 11 inflammatory markers. Analysis by PCA revealed the collective importance of this cytokine signature in associations with disease severity, need for invasive mechanical ventilation and mortality. These findings characterise a distinct pro-inflammatory signature in severe COVID-19, they add further evidence to support a moderate, but distinct, cytokine elevation in severe COVID-19. In sum, our findings further validate previous studies and highlight a severe pro-inflammatory phenotype and distinct cytokine profile in severe COVID-19 (25).

## Data availability statement

The raw data supporting the conclusions of this article will be made available by the authors, without undue reservation.

## Ethics statement

The studies involving human participants were reviewed and approved by St James’s Hospital/Tallaght University Hospital Joint Research Ethics Committee. The patients/participants provided their written informed consent to participate in this study.

## Author contributions

Overall study design: HH, NP, NO’D, LO’D, AO’H, RK, ML, IM-L, CB, AL, NC, CNC, DD, and PF. Participant recruitment and assessment: AD, AUM, AG, CR, SD, SPK, JS, and STTAR COVID-19 Bioresource. Experiments: HH, AD, NP, NO’D, and PF. Curation of Data: HH, AD, NP, NO’D, LO’D, AG, and JS. Analysis and Visualisation of Data: AD, MM, NB, SPK, and PF. Manuscript Preparation: AD and PF. All authors contributed to the article and approved the submitted version.

## References

[1] Zhou F Yu T Du R Fan G Liu Y Liu Z. Clinical course and risk factors for mortality of adult inpatients with COVID-19 in wuhan, China: A retrospective cohort study. Lancet (2020) 395(10229):1054–62. doi: 10.1016/S0140-6736(20)30566-3 PMC7270627 32171076

[2] Dessie ZG Zewotir T. Mortality-related risk factors of COVID-19: A systematic review and meta-analysis of 42 studies and 423,117 patients. BMC Infect Dis (2021) 21(1):855. doi: 10.1186/s12879-021-06536-3 34418980 PMC8380115

[3] Welch C. Age and frailty are independently associated with increased COVID-19 mortality and increased care needs in survivors: Results of an international multi-centre study. Age Ageing (2021) 50(3):617–30. doi: 10.1093/ageing/afab026 PMC7929433 33543243

[4] Merad M Blish CA Sallusto F Iwasaki A. The immunology and immunopathology of COVID-19. Science (2022) 375(6585):1122–7. doi: 10.1126/science.abm8108 PMC12828912 35271343

[5] Merad M Subramanian A Wang TT. An aberrant inflammatory response in severe COVID-19. Cell Host Microbe (2021) 29(7):1043–7. doi: 10.1016/j.chom.2021.06.018 PMC8279571 34265243

[6] Lucas C Wong P Klein J Castro TBR Silva J Sundaram M. Longitudinal analyses reveal immunological misfiring in severe COVID-19. Nature (2020) 584(7821):463–9. doi: 10.1038/s41586-020-2588-y PMC7477538 32717743

[7] O'Driscoll DN. Emergency myelopoiesis in critical illness: lessons from the COVID-19 pandemic. Ir J Med Sci (2022) 16:1–2. doi: 10.1007/s11845-022-03068-w PMC9203045 35711011

[8] Townsend L Dyer AH Naughton A Imangaliyev S Dunne J Kiersey R. Severe COVID-19 is characterised by inflammation and immature myeloid cells early in disease progression. Heliyon (2022) 8(4):e09230. doi: 10.1016/j.heliyon.2022.e09230 35386227 PMC8973020

[9] Asano T Boisson B Onodi F Matuozzo D Moncada-Velez M Maglorius Renkilaraj MRL. X-Linked recessive TLR7 deficiency in ~1% of men under 60 years old with life-threatening COVID-19. Sci Immunol (2021) 6(62). doi: 10.1126/sciimmunol.abl4348 PMC8532080 34413140

[10] Bastard P Gervais A Le Voyer T Rosain J Philippot Q Manry J. Autoantibodies neutralizing type I IFNs are present in ~4% of uninfected individuals over 70 years old and account for ~20% of COVID-19 deaths. Sci Immunol (2021) 6(62). doi: 10.1126/sciimmunol.abl4340 PMC8521484 34413139

[11] Manry J Bastard P Gervais A Le Voyer T Rosain J Philippot Q. The risk of COVID-19 death is much greater and age dependent with type I IFN autoantibodies. Proc Natl Acad Sci U S A (2022) 119(21):e2200413119. doi: 10.1073/pnas.2200413119 35576468 PMC9173764

[12] Copaescu A Smibert O Gibson A Phillips EJ Trubiano JA. The role of IL-6 and other mediators in the cytokine storm associated with SARS-CoV-2 infection. J Allergy Clin Immunol (2020) 146(3):518–34.e1. doi: 10.1016/j.jaci.2020.07.001 32896310 PMC7471766

[13] Mudd PA Crawford JC Turner JS Souquette A Reynolds D Bender D. Distinct inflammatory profiles distinguish COVID-19 from influenza with limited contributions from cytokine storm. Sci Adv (2020) 6(50). doi: 10.1126/sciadv.abe3024 PMC7725462 33187979

[14] Mueller AA Tamura T Crowley CP DeGrado JR Haider H Jezmir JL. Inflammatory biomarker trends predict respiratory decline in COVID-19 patients. Cell Rep Med (2020) 1(8):100144. doi: 10.1016/j.xcrm.2020.100144 33163981 PMC7598305

[15] Sinha P Calfee CS Cherian S Brealey D Cutler S King C. Prevalence of phenotypes of acute respiratory distress syndrome in critically ill patients with COVID-19: a prospective observational study. Lancet Respir Med (2020) 8(12):1209–18. doi: 10.1016/S2213-2600(20)30366-0 PMC7718296 32861275

[16] Recovery Collaborative Group. Tocilizumab in patients admitted to hospital with COVID-19 (RECOVERY): a randomised, controlled, open-label, platform trial. Lancet (2021) 397(10285):1637–45. doi: 10.1016/S0140-6736(21)00676-0 PMC8084355 33933206

[17] Broman N Feuth T Vuorinen T Valtonen M Hohenthal U Löyttyniemi E. Early administration of tocilizumab in hospitalized COVID-19 patients with elevated inflammatory markers; COVIDSTORM-a prospective, randomized, single-centre, open-label study. Clin Microbiol Infect (2022) 28(6):844–51. doi: 10.1016/j.cmi.2022.02.027 PMC8897958 35259529

[18] Gordon AC Mouncey PR Al-Beidh F Rowan KM Nichol AD Arabi YM. Interleukin-6 receptor antagonists in critically ill patients with covid-19. N Engl J Med (2021) 384(16):1491–502. doi: 10.1056/NEJMoa2100433 PMC7953461 33631065

[19] Rosas IO Bräu N Waters M Go RC Hunter BD Bhagani S. Tocilizumab in hospitalized patients with severe covid-19 pneumonia. N Engl J Med (2021) 384(16):1503–16. doi: 10.1056/NEJMoa2028700 PMC7953459 33631066

[20] Makaremi S Asgarzadeh A Kianfar H Mohammadnia A Asghariazar V Safarzadeh E. The role of IL-1 family of cytokines and receptors in pathogenesis of COVID-19. Inflammation Res (2022) 71(7-8):923–47. doi: 10.1007/s00011-022-01596-w PMC9243884 35751653

[21] Hasegawa T Hato T Okayama T Ikeo K Miyamoto Y Iwanaga N. Th1 cytokine endotype discriminates and predicts severe complications in COVID-19. Eur Cytokine Netw (2022) 33(2):1–12. doi: 10.1684/ecn.2022.0477 36266985 PMC9595088

[22] Gibellini L De Biasi S Meschiari M Gozzi L Paolini A Borella R. Plasma cytokine atlas reveals the importance of TH2 polarization and interferons in predicting COVID-19 severity and survival. Front Immunol (2022) 13:842150. doi: 10.3389/fimmu.2022.842150 35386702 PMC8979161

[23] Pourgholaminejad A Pahlavanneshan S Basiri M. COVID-19 immunopathology with emphasis on Th17 response and cell-based immunomodulation therapy: Potential targets and challenges. Scand J Immunol (2022) 95(2):e13131. doi: 10.1111/sji.13131 34936112

[24] Khalil BA Elemam NM Maghazachi AA. Chemokines and chemokine receptors during COVID-19 infection. Comput Struct Biotechnol J (2021) 19:976–88. doi: 10.1016/j.csbj.2021.01.034 PMC7859556 33558827

[25] Del Valle DM Kim-Schulze S Huang HH Beckmann ND Nirenberg S Wang B. An inflammatory cytokine signature predicts COVID-19 severity and survival. Nat Med (2020) 26(10):1636–43. doi: 10.1038/s41591-020-1051-9 PMC7869028 32839624

[26] Chi Y Ge Y Wu B Zhang W Wu T Wen T. Serum cytokine and chemokine profile in relation to the severity of coronavirus disease 2019 in China. J Infect Dis (2020) 222(5):746–54. doi: 10.1093/infdis/jiaa363 PMC7337752 32563194

[27] Guo J Wang S Xia H Shi D Chen Y Zheng S. Cytokine signature associated with disease severity in COVID-19. Front Immunol (2021) 12:681516. doi: 10.3389/fimmu.2021.681516 34489933 PMC8418386

[28] Guo Y Li T Xia X Su B Li H Feng Y. Different profiles of antibodies and cytokines were found between severe and moderate COVID-19 patients. Front Immunol (2021) 12:723585. doi: 10.3389/fimmu.2021.723585 34489974 PMC8417126

[29] Han H Ma Q Li C Liu R Zhao L Wang W. Profiling serum cytokines in COVID-19 patients reveals IL-6 and IL-10 are disease severity predictors. Emerg Microbes Infect (2020) 9(1):1123–30. doi: 10.1080/22221751.2020.1770129 PMC7473317 32475230

[30] McElvaney OJ Hobbs BD Qiao D McElvaney OF Moll M McEvoy NL. A linear prognostic score based on the ratio of interleukin-6 to interleukin-10 predicts outcomes in COVID-19. EBioMedicine (2020) 61:103026. doi: 10.1016/j.ebiom.2020.103026 33039714 PMC7543971

[31] Jia F Wang G Xu J Long J Deng F Jiang W. Role of tumor necrosis factor-α in the mortality of hospitalized patients with severe and critical COVID-19 pneumonia. Aging (Albany NY) (2021) 13(21):23895–912. doi: 10.18632/aging.203663 PMC8610114 34725309

[32] Messing M Sekhon MS Hughes MR Stukas S Hoiland RL Cooper J. Prognostic peripheral blood biomarkers at ICU admission predict COVID-19 clinical outcomes. Front Immunol (2022) 13:1010216. doi: 10.3389/fimmu.2022.1010216 36451808 PMC9703061

[33] O'Doherty L Hendricken Phelan S Wood N O'Brien S Sui J Mangan C. Study protocol for the St james's hospital, tallaght university hospital, trinity college Dublin allied researchers' (STTAR) bioresource for COVID-19. HRB Open Res (2022) 5:20. doi: 10.12688/hrbopenres.13498.1 35615437 PMC9111362

[34] Rockwood K Song X MacKnight C Bergman H Hogan DB McDowell I. A global clinical measure of fitness and frailty in elderly people. Cmaj (2005) 173(5):489–95. doi: 10.1503/cmaj.050051 PMC1188185 16129869

[35] WHO Working Group on the Clinical Characterisation and Management of COVID-19 Infection. A minimal common outcome measure set for COVID-19 clinical research. Lancet Infect Dis (2020) 20(8):e192–e7. doi: 10.1016/S1473-3099(20)30483-7 PMC7292605 32539990

[36] Mehta P Fajgenbaum DC. Is severe COVID-19 a cytokine storm syndrome: a hyperinflammatory debate. Curr Opin Rheumatol (2021) 33(5):419–30. doi: 10.1097/BOR.0000000000000822 PMC8373392 34264880

[37] Yang Y Shen C Li J Yuan J Wei J Huang F. Plasma IP-10 and MCP-3 levels are highly associated with disease severity and predict the progression of COVID-19. J Allergy Clin Immunol (2020) 146(1):119–27.e4. doi: 10.1016/j.jaci.2020.04.027 32360286 PMC7189843

[38] Lev S Gottesman T Sahaf Levin G Lederfein D Berkov E Diker D. Observational cohort study of IP-10's potential as a biomarker to aid in inflammation regulation within a clinical decision support protocol for patients with severe COVID-19. PloS One (2021) 16(1):e0245296. doi: 10.1371/journal.pone.0245296 33434221 PMC7802954

[39] Chen Y Wang J Liu C Su L Zhang D Fan J. IP-10 and MCP-1 as biomarkers associated with disease severity of COVID-19. Mol Med (2020) 26(1):97. doi: 10.1186/s10020-020-00230-x 33121429 PMC7594996

[40] Lorè NI De Lorenzo R Rancoita PMV Cugnata F Agresti A Benedetti F. CXCL10 levels at hospital admission predict COVID-19 outcome: hierarchical assessment of 53 putative inflammatory biomarkers in an observational study. Mol Med (2021) 27(1):129. doi: 10.1186/s10020-021-00390-4 34663207 PMC8521494

[41] Jøntvedt Jørgensen M Holter JC Christensen EE Schjalm C Tonby K Pischke SE. Increased interleukin-6 and macrophage chemoattractant protein-1 are associated with respiratory failure in COVID-19. Sci Rep (2020) 10(1):21697. doi: 10.1038/s41598-020-78710-7 33303843 PMC7729930

[42] Burke H Freeman A Cellura DC Stuart BL Brendish NJ Poole S. Inflammatory phenotyping predicts clinical outcome in COVID-19. Respir Res (2020) 21(1):245. doi: 10.1186/s12931-020-01511-z 32962703 PMC7506817

[43] Furci F Murdaca G Allegra A Gammeri L Senna G Gangemi S. IL-33 and the cytokine storm in COVID-19: From a potential immunological relationship towards precision medicine. Int J Mol Sci (2022) 23(23). doi: 10.3390/ijms232314532 PMC9740753 36498859

[44] Gao Y Cai L Li L Zhang Y Li J Luo C. Emerging effects of IL-33 on COVID-19. Int J Mol Sci (2022) 23(21). doi: 10.3390/ijms232113656 PMC9658128 36362440

[45] Stanczak MA Sanin DE Apostolova P Nerz G Lampaki D Hofmann M. IL-33 expression in response to SARS-CoV-2 correlates with seropositivity in COVID-19 convalescent individuals. Nat Commun (2021) 12(1):2133. doi: 10.1038/s41467-021-22449-w 33837219 PMC8035172

[46] Fajgenbaum DC June CH. Cytokine storm. N Engl J Med (2020) 383(23):2255–73. doi: 10.1056/NEJMra2026131 PMC7727315 33264547

[47] Leisman DE Ronner L Pinotti R Taylor MD Sinha P Calfee CS. Cytokine elevation in severe and critical COVID-19: a rapid systematic review, meta-analysis, and comparison with other inflammatory syndromes. Lancet Respir Med (2020) 8(12):1233–44. doi: 10.1016/S2213-2600(20)30404-5 PMC7567529 33075298

[48] Olbei M Hautefort I Modos D Treveil A Poletti M Gul L. SARS-CoV-2 causes a different cytokine response compared to other cytokine storm-causing respiratory viruses in severely ill patients. Front Immunol (2021) 12:629193. doi: 10.3389/fimmu.2021.629193 33732251 PMC7956943

[49] Sims JT Krishnan V Chang CY Engle SM Casalini G Rodgers GH. Characterization of the cytokine storm reflects hyperinflammatory endothelial dysfunction in COVID-19. J Allergy Clin Immunol (2021) 147(1):107–11. doi: 10.1016/j.jaci.2020.08.031 PMC7488591 32920092

[50] Wilson JG Simpson LJ Ferreira AM Rustagi A Roque J Asuni A. Cytokine profile in plasma of severe COVID-19 does not differ from ARDS and sepsis. JCI Insight (2020) 5(17). doi: 10.1172/jci.insight.140289 PMC7526438 32706339

[51] Öcal S. SARS-CoV-2 and lung injury: Dysregulation of immune response but not hyperimmune response as in "cytokine storm syndrome". Clin Respir J (2022) 16(1):13–6. doi: 10.1111/crj.13455 PMC8653083 34674363

[52] Cron RQ Caricchio R Chatham WW. Calming the cytokine storm in COVID-19. Nat Med (2021) 27(10):1674–5. doi: 10.1038/s41591-021-01500-9 34480126

[53] Bonnet B Cosme J Dupuis C Coupez E Adda M Calvet L. Severe COVID-19 is characterized by the co-occurrence of moderate cytokine inflammation and severe monocyte dysregulation. EBioMedicine (2021) 73:103622. doi: 10.1016/j.ebiom.2021.103622 34678611 PMC8526358

[54] Wang SY Takahashi T Pine AB Damsky WE Simonov M Zhang Y. Challenges in interpreting cytokine data in COVID-19 affect patient care and management. PloS Biol (2021) 19(8):e3001373. doi: 10.1371/journal.pbio.3001373 34358229 PMC8372945

[55] Knight V Long T Meng QH Linden MA Rhoads DD. Variability in the laboratory measurement of cytokines. Arch Pathol Lab Med (2020) 144(10):1230–3. doi: 10.5858/arpa.2019-0519-CP 32401053

[56] Sinha S Rosin NL Arora R Labit E Jaffer A Cao L. Dexamethasone modulates immature neutrophils and interferon programming in severe COVID-19. Nat Med (2022) 28(1):201–11. doi: 10.1038/s41591-021-01576-3 PMC8799469 34782790

[57] Xu X Han M Li T Sun W Wang D Fu B. Effective treatment of severe COVID-19 patients with tocilizumab. Proc Natl Acad Sci U S A (2020) 117(20):10970–5. doi: 10.1073/pnas.2005615117 PMC7245089 32350134

[58] Smith N Possémé C Bondet V Sugrue J Townsend L Charbit B. Defective activation and regulation of type I interferon immunity is associated with increasing COVID-19 severity. Nat Commun (2022) 13(1):7254. doi: 10.1038/s41467-022-34895-1 36434007 PMC9700809

[59] Reddy K Sinha P O'Kane CM Gordon AC Calfee CS McAuley DF. Subphenotypes in critical care: translation into clinical practice. Lancet Respir Med (2020) 8(6):631–43. doi: 10.1016/S2213-2600(20)30124-7 32526190

