# Supplementary material for: Characterisation of the pro-inflammatory cytokine signature in severe COVID-19
Source: Front Immunol. 2023 Mar 30;14:1170012. doi: 10.3389/fimmu.2023.1170012 (PMC10101230; doi:10.3389/fimmu.2023.1170012)
Supplement: Supplementary file 1 [file DataSheet_1.docx]

**Table S1. Inflammatory Markers Differing By COVID-19 Status in Participants with Acute COVID-19 Illness (N = 118) vs Healthy Controls (N = 44)**

| **Marker** | **HC Undetectable**  **N (%)** | **COVID-19 Undetectable**  **N (%)** | **HC**  **Median conc. (IQR)** | **COVID-19**  **Median conc. (IQR)** | **Statistic** |
| --- | --- | --- | --- | --- | --- |
| IL-1β (fg/mL) | 1 (2.2%) | 3 (2.5%) | 87.7  (53.5-226.1) | 107.3  (54.5-280) | z = -0.6, p = 0.53 |
| IL-2 (fg/mL) | 1 (2.2%) | 0 (0%) | 41.8  (26.8-59.8) | 207.2  (90.4-344.4) | z = -7.91, p<0.001 |
| IL-4 (fg/mL) | 1 (2.2%) | 3 (2.5%) | 16.1  (10.3-24.9) | 16.4  (7.6-33.1) | z = -0.1, p = 0.93 |
| IL-5 (pg/mL) | 37 (85%) | 30 (25%) | 0.2  (0.1-0.8) | 0.6  (0.3-1.1) | z = -2.0, p = 0.04 |
| IL-6 (fg/mL) | 1 (2.2%) | 0 (0%) | 832.4  (617.0-1484.1) | 9,214.7  (3,785.1-26,994.0) | z = -7.9, p<0.001 |
| IL-10 (fg/mL) | 1 (2.2%) | 0 (0%) | 237.0  (181.8-425.9) | 2,054.3  (816.8-5,413.5) | z = -8.6, p<0.001 |
| IL-12p70 (fg/mL) | 1 (2.2%) | 0 (0%) | 151.6  (85.8-233.6) | 143.5  (79.5-239.3) | z = 0.1, p = 0.90 |
| IL-13 (pg/mL) | 28 (63.6%) | 57 (48.3%) | 1.02  (0.65-1.66) | 10.2  (6.1-14.6) | z = -5.5, p<0.001 |
| IL-17A (fg/mL) | 1 (2.2%) | 0 (0%) | 161.2  (108.3-393.8) | 388.9  (170.5-973.9) | z = -3.1, p=0.02 |
| IL-18 (pg/mL) | 0 (0%) | 0 (0%) | 269.8  (197.4-371.7) | 569.8  (382.9-889.0) | z = -5.8, p<0.001 |
| IL-23 (pg/mL) | 31 (70.5%) | 0 (0%) | 0.3  (0.1-0.5) | 4.2  (2.3-6.2) | z = -5.8, p<0.001 |
| IL-33 (pg/mL) | 39 (88.6%) | 3 (2.5%) | 1.14  (0.40-1.71) | 2.8  (1.7-4.7) | z = -2.6, p=0.001 |
| TNF-α (fg/mL) | 1 (2.2%) | 0 (0%) | 349.8  (281.9-476.4) | 814.4  (590.2-1,324) | z = -7.6, p<0.001 |
| IP-10 (pg/mL) | 0 (0%) | 0 (0%) | 129.8  (88.2-188.6) | 761.4  (313.9-2,161.2) | z = -8.1, p<0.001 |
| IFN-y (fg/mL) | 1 (2.2%) | 0 (0%) | 286.3  (172.6-434.8) | 793.7  (298.2-2,545.5) | z = -4.5, p<0.001 |
| MCP-1(pg/mL) | 0 (0%) | 0 (0%) | 97.8  (64.2-132.3) | 166.1  (109.8-256.9) | z = -5.5, p<0.001 |
| MIP-1β (pg/mL) | 0 (0%) | 0 (0%) | 23.9  (17.9-34.8) | 44.7  (30.4-65.0) | z = -6.0, p<0.001 |
| G-CSF (pg/mL) | 9 (20.5%) | 0 (0%) | 1.1  (0.6-2.1) | 13.2  (8.7-21.8) | z = -8.8, p<0.001 |
| TGF-β (pg/mL) | 0 (0%) | 7 (6%) | 8,061.1  (3,602-17,573) | 3,761  (2,054-8,331) | z = 3.1, p=0.002 |
| YKL-40 (pg/mL) | 0 (0%) | 0 (0%) | 19,492  (11,520-35,102) | 85,198  (35,763-189,320) | z = -6.3, p<0.001 |

**Table S2. Inflammatory Markers Differing By COVID-19 Severity in Participants with Acute COVID-19 Illness (N = 118)**

| **Marker** | **Mild (N = 26; 16.1%)**  **Median conc. (IQR)** | **Moderate (N = 58; 35.8%)**  **Median conc. (IQR)** | **Severe (N = 34; 21.0%)**  **Median conc. (IQR)** | **Statistic** |
| --- | --- | --- | --- | --- |
| IL-1β (fg/mL) | 151.7  (74.7-321.2) | 87.7  (48.9-201.4) | 152.92  (80-514.7) | χ^2^ = 8, p = 0.02 |
| IL-2 (fg/mL) | 141.8  (88.9-238.6) | 154.2  (68.5-269.4) | 341.2  (215.7-492.8) | χ^2^ = 15.8, p = 0.0004 |
| IL-4 (fg/mL) | 18.4  (8.6-32.6) | 15.5  (6.9-26.6) | 16.1  (7.0-47.6) | χ^2^ = 1.4, p = 0.50 |
| IL-5 (pg/mL) | 0.9  (0.4-1.3) | 0.6  (0.3-0.9) | 0.6  (0.3-1.2) | χ^2^ = 1.7, p = 0.42 |
| IL-6 (fg/mL) | 9,229.7  (3,199.8-17,069.1) | 5,817.8  (2,484.8-17,208.7) | 18,897  (9,602.6-57,376.8) | χ^2^ = 17.1, p = 0.0002 |
| IL-10 (fg/mL) | 882.2  (637.3-2,137.4) | 1,864.5  (865.1-4.824.2) | 4,760.5  (1855.2-8867.9) | χ^2^ = 15.9, p = 0.0002 |
| IL-12p70 (fg/mL) | 154.5  (132.3-242.7) | 114.1  (84.1-197.0) | 165.3  (74.4-32.7) | χ^2^ = 2.3, p = 0.32 |
| IL-13 (pg/mL) | 11.1  (5.3-13.5) | 9.7  (5.1-14.6) | 10.1  (7.0-15.0) | χ^2^ = 0.5, p = 0.81 |
| IL-17A (fg/mL) | 432.2  (254.7-1131.9) | 383.2  (120.9-874.5) | 386.1  (176.8-912.6) | χ^2^ = 1.3, p = 0.53 |
| IL-18 (pg/mL) | 530.9  (357.7-672.3) | 530.5  (382.9-753.9) | 767.8  (560.0-1125.2) | χ^2^ = 8.5, p = 0.01 |
| IL-23 (pg/mL) | 3.1  (1.6-4.6) | 4.1  (2.8-6.2) | 5.8  (3.2-4.9) | χ^2^ = 9.5, p = 0.01 |
| IL-33 (pg/mL) | 2.0  (1.0-3.3) | 2.7  (1.8-4.2) | 4.9  (2.4-6.3) | χ^2^ = 12.6, p = 0.002 |
| TNF-α (fg/mL) | 951.4  (600.7-1410.8) | 735.3  (485.2-1126.9) | 951.5  (699.0-1500.9) | χ^2^ = 7.7, p = 0.02 |
| IP-10 (pg/mL) | 486.2  (292.5-909.3) | 637.2  (286.9-2,523.9) | 1,224.7  (668.1-2,741.9) | χ^2^ = 11.9, p = 0.004 |
| IFN-y (fg/mL) | 1109.6  (520.9-2580.5) | 793.7  (264.1-2,456.9) | 726.9  (239.3-2,545.5) | χ^2^ = 1.7, p = 0.44 |
| MCP-1(pg/mL) | 162.6  (132.7-200.9) | 149.1  (97.1-235.7) | 194.3  (135.6-289.0) | χ^2^ = 4.3, p = 0.12 |
| MIP-1β (pg/mL) | 45.2  (30.5-57.6) | 38.41  (28.1-62.9) | 50.5  (39.9-65.6) | χ^2^ = 4.1, p = 0.13 |
| G-CSF (pg/mL) | 11.2  (8.30-14.7) | 16.9  (8.5-24.4) | 13.4  (11.8-24.1) | χ^2^ = 6.2, p = 0.05 |
| TGF-β (pg/mL) | 4,237.4  (2,394.4-8,575.0) | 4,156.5  (2,100.4-11,062.9) | 3,321.6  (1,945.2-6,560.5) | χ^2^ = 2.3, p = 0.31 |
| YKL-40 (pg/mL) | 91,615  (30,274-157,617) | 71,442  (29,803-134,918) | 133,476  (56,684-248,128) | χ^2^ = 7.3, p = 0.03 |

**Table S3. Correlation Matrix of Inflammatory Markers Differing in Participants with Acute COVID-19 Illness (N = 118).** Values in bold font indicate significant pairwise comparisons at p<0.05. There were low-modest correlations for most markers. Strongest correlations were seen (R>0.7) between 6 pairs: IL-1β & IL-4, IL-1β & TNF**α**, IL-1β & IL-6, between IL-6 & TNF**α**; between TNF**α** and IL-10 and between IP-10 & IL-33 which all persisted following Bonferonni correction.

|  | **IL1β** | **IL2** | **IL4** | **IL5** | **IL6** | **IL10** | **IL12p70** | **IL13** | **IL17A** | **IL18** | **IL23** | **IL33** | **TNF-α** | **IP10** | **IFNy** | **MCP1** | **MIP1β** | **GCSF** | **TGF-β** | **YKL-40** |
| --- | --- | --- | --- | --- | --- | --- | --- | --- | --- | --- | --- | --- | --- | --- | --- | --- | --- | --- | --- | --- |
| **IL1β** | **1.00** |  |  |  |  |  |  |  |  |  |  |  |  |  |  |  |  |  |  |  |
| **IL2** | **0.54** | **1.00** |  |  |  |  |  |  |  |  |  |  |  |  |  |  |  |  |  |  |
| **IL4** | **0.71** | **0.55** | **1.00** |  |  |  |  |  |  |  |  |  |  |  |  |  |  |  |  |  |
| **IL5** | 0.07 | 0.03 | 0.02 | **1.00** |  |  |  |  |  |  |  |  |  |  |  |  |  |  |  |  |
| **IL6** | **0.77** | **0.70** | **0.62** | -0.10 | **1.00** |  |  |  |  |  |  |  |  |  |  |  |  |  |  |  |
| **IL10** | **0.33** | **0.69** | **0.34** | -0.02 | **0.54** | **1.00** |  |  |  |  |  |  |  |  |  |  |  |  |  |  |
| **IL12p70** | **0.48** | **0.56** | **0.68** | 0.05 | **0.45** | 0.29 | **1.00** |  |  |  |  |  |  |  |  |  |  |  |  |  |
| **IL13** | -0.09 | -0.02 | -0.23 | 0.27 | -0.04 | 0.00 | -0.07 | **1.00** |  |  |  |  |  |  |  |  |  |  |  |  |
| **IL17A** | **0.45** | **0.40** | **0.47** | 0.17 | **0.47** | 0.09 | **0.44** | -0.12 | **1.00** |  |  |  |  |  |  |  |  |  |  |  |
| **IL18** | **0.50** | **0.40** | **0.44** | -0.09 | **0.42** | **0.40** | 0.31 | -0.06 | 0.17 | **1.00** |  |  |  |  |  |  |  |  |  |  |
| **IL23** | **0.37** | **0.45** | 0.24 | 0.16 | **0.44** | **0.39** | 0.15 | 0.25 | 0.17 | **0.46** | **1.00** |  |  |  |  |  |  |  |  |  |
| **IL33** | **0.36** | **0.53** | 0.30 | 0.04 | **0.47** | **0.54** | 0.24 | 0.21 | 0.05 | **0.34** | **0.65** | **1.00** |  |  |  |  |  |  |  |  |
| **TNF-α** | **0.72** | **0.67** | **0.69** | 0.16 | **0.75** | **0.44** | **0.61** | -0.06 | **0.62** | **0.37** | 0.33 | **0.39** | **1.00** |  |  |  |  |  |  |  |
| **IP10** | **0.42** | **0.69** | **0.44** | -0.10 | **0.60** | **0.75** | **0.35** | 0.12 | 0.02 | **0.45** | **0.51** | **0.75** | **0.47** | **1.00** |  |  |  |  |  |  |
| **IFNy** | **0.55** | **0.65** | **0.67** | 0.12 | **0.55** | **0.60** | **0.50** | -0.09 | **0.34** | **0.39** | **0.34** | **0.49** | **0.59** | **0.64** | 1.00 |  |  |  |  |  |
| **MCP1** | **0.45** | **0.48** | **0.43** | 0.04 | **0.52** | 0.32 | 0.32 | -0.05 | 0.33 | 0.21 | 0.13 | 0.29 | **0.59** | **0.41** | **0.43** | 1.00 |  |  |  |  |
| **MIP1β** | **0.44** | 0.32 | 0.16 | 0.13 | **0.33** | 0.31 | 0.14 | -0.02 | 0.13 | **0.35** | **0.35** | **0.35** | **0.43** | 0.29 | 0.18 | 0.30 | 1.00 |  |  |  |
| **GCSF** | 0.28 | **0.40** | 0.27 | 0.11 | **0.40** | **0.40** | 0.18 | 0.14 | 0.25 | 0.19 | **0.46** | **0.43** | 0.30 | **0.50** | **0.48** | 0.32 | 0.03 | 1.00 |  |  |
| **TGFβ** | -0.07 | -0.13 | -0.09 | 0.02 | -0.21 | -0.11 | -0.02 | -0.01 | -0.01 | -0.01 | -0.08 | -0.06 | -0.19 | -0.10 | -0.16 | -0.15 | -0.05 | -0.07 | 1.00 |  |
| **YKL-40** | **0.39** | **0.43** | 0.31 | 0.16 | **0.53** | 0.31 | 0.34 | -0.02 | **0.42** | 0.34 | **0.34** | 0.23 | **0.47** | 0.29 | 0.18 | 0.30 | 0.21 | 0.27 | -0.06 | 1.00 |

**Figure S1**

**
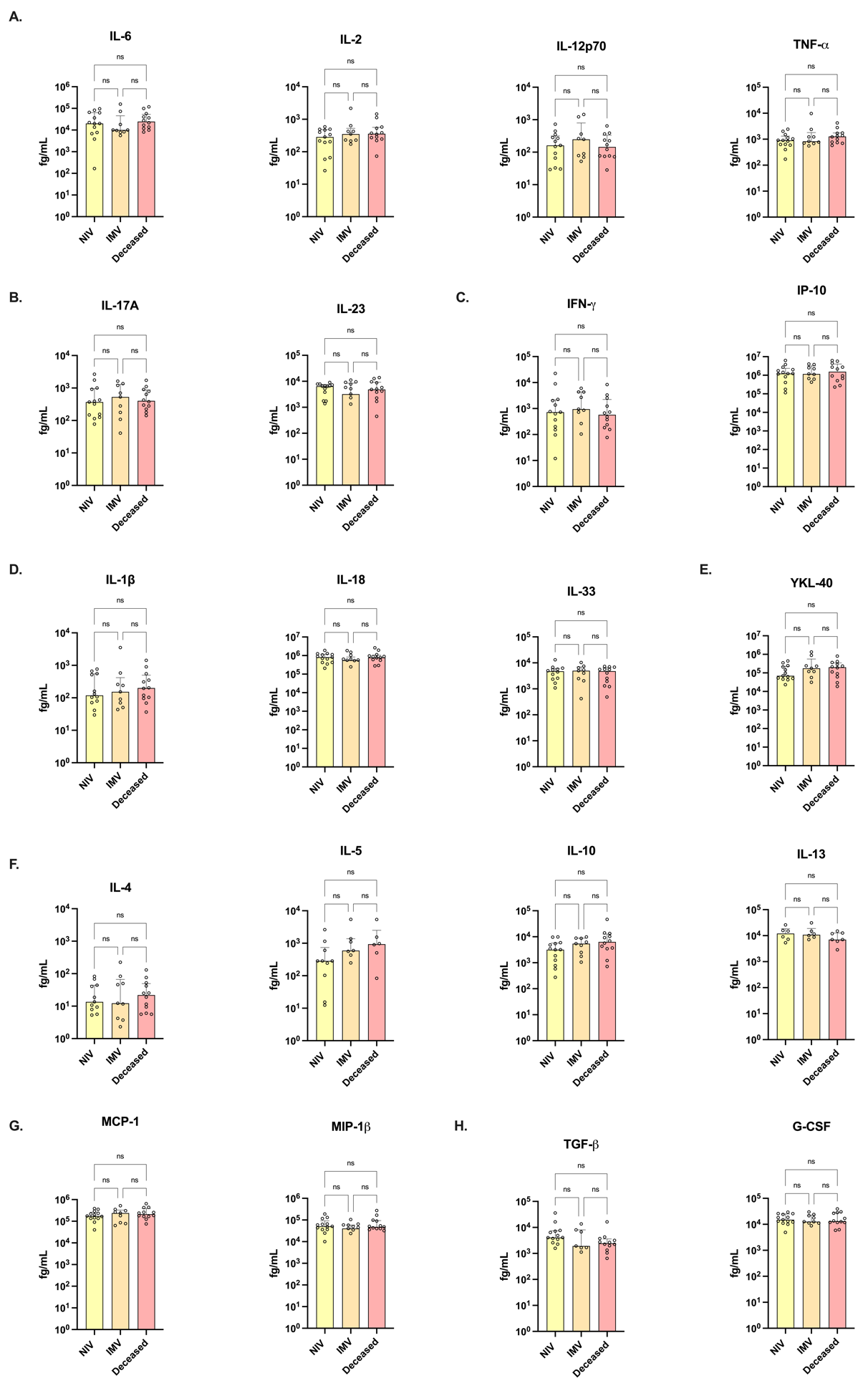
**

**Figure 1. Cytokine Concentrations in Severe COVID-19**. Markers of Th1 (A), Th17 (B), Interferon (C), IL-1 family (D), YKL-40 (E), Th2 (F), Chemokine (G) and Growth Factor (H) Responses Did Not Significantly Differ Between Individuals Requiring Non-Invasive Ventilation (WHO Score 6), Invasive Mechanical Ventilation (WHO Scores: 7-9) and those who Died (WHO Score 10). IMV: Invasive Mechanical Ventilation, NIV: Non-Invasive Mechanical Ventilation; ns = not significant
